# Genotyping of SNPs in bread wheat at reduced cost from pooled experiments and imputation

**DOI:** 10.1007/s00122-023-04533-5

**Published:** 2024-01-19

**Authors:** Camille Clouard, Carl Nettelblad

**Affiliations:** 1 Division of Scientific Computing, Department of Information Technology, Uppsala University, Lägerhyddsvägen 1, 75237 Uppsala, Sweden; 2 SciLifeLab, Science for Life Laboratory, Husargatan 3, 75237 Uppsala, Sweden

## Abstract

**Key message:**

Pooling and imputation are computational methods that can be combined for achieving cost-effective and accurate high-density genotyping of both common and rare variants, as demonstrated in a MAGIC wheat population.

**Abstract:**

The plant breeding industry has shown growing interest in using the genotype data of relevant markers for performing selection of new competitive varieties. The selection usually benefits from large amounts of marker data, and it is therefore crucial to dispose of data collection methods that are both cost-effective and reliable. Computational methods such as genotype imputation have been proposed earlier in several plant science studies for addressing the cost challenge. Genotype imputation methods have though been used more frequently and investigated more extensively in human genetics research. The various algorithms that exist have shown lower accuracy at inferring the genotype of genetic variants occurring at low frequency, while these rare variants can have great significance and impact in the genetic studies that underlie selection. In contrast, pooling is a technique that can efficiently identify low-frequency items in a population, and it has been successfully used for detecting the samples that carry rare variants in a population. In this study, we propose to combine pooling and imputation and demonstrate this by simulating a hypothetical microarray for genotyping a population of recombinant inbred lines in a cost-effective and accurate manner, even for rare variants. We show that with an adequate imputation model, it is feasible to accurately predict the individual genotypes at lower cost than sample-wise genotyping and time-effectively. Moreover, we provide code resources for reproducing the results presented in this study in the form of a containerized workflow.

## Introduction

Genotype imputation is a common computational strategy that is adopted for augmenting the density of lab-determined genotypes. Imputation is a standard practice with human data, for which several accurate and efficient imputation algorithms have been developed and tested, as well as large reference panels are available (Das et al. 2018). In crop species, genotype imputation represents a promising and relevant computational technique for producing data supporting the decisions for conducting selection cycles in plant breeding (Skøt and Grinberg 2017; Rasheed and Xia 2019; Maccaferri et al. 2022). Genotype imputation can be performed with array-based genotype data, but it is also a particularly valuable method for processing genotyping-by-sequencing (GBS) data in which there is often a high missing rate of genotypes, especially in the case of low-coverage sequencing which requires specific treatment (Fragoso et al. 2015; Zheng et al. 2018). Some methods originally designed for imputation in outbred human populations, for instance Beagle, have shown high accuracy in wheat and other crop species, under the condition of adapting the default parameters (Pook 2019). Indeed, plant breeders often use experimental crop populations that involve extensive pedigrees and deliberate selfing of individuals into pure lines. Examples of this can include multi-parent advanced generation intercross (MAGIC) populations. The selfing phenomenon is not encountered in the human species, such that at the cohort level, the characteristics of a natural human population might substantially differ from a MAGIC crop population.

The specific genetic structure of experimental plant populations can pose challenges that need to be addressed by implementing tailored imputation methods, in order to allow for the practical use of high-accuracy genotype imputation in plant breeding. First, inbred populations typically have a low level of genetic diversity and second, a very high proportion of fully homozygous individuals. Some specific imputation methods have taken advantage of these features (Pook 2019; Gonen et al. 2018). Moreover, the algorithms and software for plant populations should accommodate relatively scarce reference data. Indeed, despite the recent efforts in developing informative resources for plant genetic research and various plant species (Gao 2020), there are rather few and small reference panels available. This can be critical since large reference panels usually improve the accuracy of imputation and the power of genome-wide association studies (GWAS). Nonetheless, using a reference panel consisting of a limited number of founder individuals can still achieve high imputation accuracy since their haplotypes represent an ideal library that suffices for explaining the genetic structure of the descendants (Fragoso et al. 2015; Thorn et al. 2021). As the data for founders can be defective or unavailable, crop scientists need imputation methods that can deal with scenarios where a reference panel other than the founders has to be used (Thorn et al. 2021). Last, crop breeding can include more dense and structured pedigrees than other populations and some imputation algorithms have exploited this characteristic (Pook 2019; Gonen et al. 2018). Still, pedigree records can turn out to be incomplete or incorrect in practice. Computational methods should therefore be able to accommodate such cases (Gonen et al. 2018).

Regardless of the species, the major weakness of reference-based genotype imputation remains the correct inference of the genotype of rare variants, even with large reference panels (Gao 2020).

Research has highlighted the great role of rare variants that can affect complex traits of agricultural relevance (Marroni et al. 2012). Inherent to their rarity, proper accounting of such variants requires the screening of even larger populations in order to detect the variants and perform statistical analyses with sufficient power.

Consequently, a main technological challenge is the ability to deliver and process large volumes of dense and accurate genotype data at a reasonable cost (Gardner et al. 2016). Pooling complemented by imputation could be a sensible strategy for meeting this challenge, with possible applications both in quantitative trait loci (QTL) analysis as well as in genomic selection (GS). We have previously demonstrated in a human setting that the composite power to detect rare variants can be improved greatly by combining pooled testing with a slightly more dense SNP chip, and imputation, rather than imputation alone with sample-wise testing on a more sparse chip (Clouard et al. 2022). It has also been proposed to combine pooling and imputation for large-scale and cost-effective application of GBS (Technow and Gerke 2017). While the interest for GBS in a plant-breeding concept is definitely increasing, breeders can still find the hybridization-based SNP arrays convenient for routine genotyping (Keeble-Gagnère et al. 2021). Arrays are also a suitable and reliable option for producing data destined for imputation since they usually yield lower missing rates and higher genotype calling accuracies (Keeble-Gagnère et al. 2021). However, despite their lower cost per data point compared to GBS, the overall cost of microarrays for single-sample genotyping can remain expensive and therefore impractical for applied usage in breeding programs. Rasheed and Xia (2019).

We propose an approach combining the usage of pooled data on a relatively dense array, that is able to directly capture rare variants, and imputation for genotyping biallelic polymorphic markers in large populations of inbred lines of bread wheat (*Triticum aestivum*). In order to demonstrate the effectiveness of our approach, we use the published genotype data from the NIAB Diverse MAGIC wheat population (Scott et al. 2021) for investigating the performance of imputation downstream to genotype pooling on a hypothetical microarray panel based on their identified subset of tag SNPs. Thanks to the processing applied to the raw reads after their original sequencing, we consider that the genotypes in the NIAB Diverse MAGIC wheat population can be used to mimic SNP chip data for a theoretical 55K array. Briefly, our pipeline simulates coalescence-based imputation from genotype probabilities that are estimated by consistently resolving the genotypes of overlapping pools of constant size. We found that an overall high genotyping accuracy of $94.5\%$ and up to $99\%$ for low-frequency variants can be achieved for a population of about 500 bread wheat accessions and roughly 1,000 SNP loci on chromosome 1A. With a pooling strategy such as the one we propose, the cost of genotyping for the whole population can be decreased since that for the same number of markers on the chip, fewer arrays are needed for genotyping pools compared to sample-wise genotyping.

## Materials and methods

From the released tag SNP genotype dataset derived through sequencing, quality control, and imputation in Scott et al. (2021), we simulate an experiment equivalent to genotyping overlapping pools of samples on microarrays, and computationally reconstructing the per-sample genotypes. The pooling approach cuts the number of microarrays but with the drawback of partially missing individual data. This caveat is addressed by imputing the genotypes with an implementation of a coalescent method on the one hand, and the Beagle software on the other. The computational performance and accuracy of the two imputation methods are compared.

**Fig. 1:**
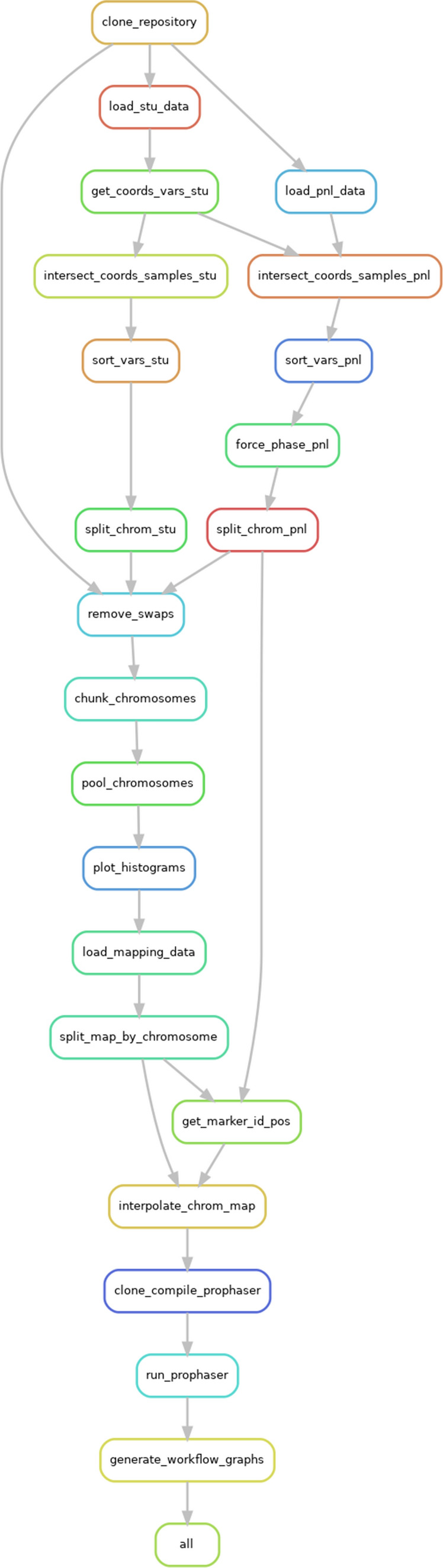
Experimental workflow Clouard (2023) Every box represents a rule that is executed by Snakemake (from top to bottom). Overall, the workflow chains three main tasks: 1. Data downloading and preprocessing: from rules "load data" to "remove swaps" and from rule "load mapping data" to "interpolate chromosome map". 2. Pooling simulation: rules "chunk chromosomes" and "pool chromosomes". 3. Imputation: rules "clone compile prophaser" and "run prophaser"

A workflow implemented with the workflow management tool *Snakemake* (Mölder et al. 2021) as well as a specific container for executing this workflow are available (Clouard 2023) if the reader would like to reproduce the results presented in this study. A fixed seed is passed to the random generator that selects the samples to be included in the study population when the workflow is executed, therefore the exact assignments of individuals to pools and other related factors should remain identical between two runs. Moreover, due to some stochasticity in the imputation algorithms, the results of imputation might also slightly differ between runs. These differences are however very small and observed for very few markers, such that the overall accuracy is not significantly affected. Figure 1 contains a Snakemake rule graph summarizing the different steps in this workflow.

### Data sources and preprocessing

In order to conduct imputation, we prepare two datasets and a genetic map for the targeted markers. The first dataset is the reference panel; the second one is the study population.

#### Genotype data of the NIAB Diverse MAGIC wheat population

We use the genotype data of an inbred population of bread wheat obtained with a MAGIC breeding scheme, which are accessible through the European Variant Archive (EVA) database. The dataset was co-published by the National Institute for Agricultural Botany (NIAB) and University College London (UCL) Genetics Institute in 2021 (Scott et al. 2021). The full wheat genome comprises three similar subgenomes A, B, and D, made of 7 pairs of chromosomes each. These subgenomes trace their origin to successive hybridizations in the development history of modern wheat.

The genotype data provided for the NIAB Diverse MAGIC (NDM) wheat population are obtained either from variant calls made in sequence data, or imputed to a large extent in the inbred lines. The founders were sequenced at high coverage, 15 of them with $\sim 23$x coverage and 1 of the founders with $\sim 16$x coverage. The inbred lines were sequenced with whole genome sequencing at low-coverage $\sim 0.3$x. The available genotype data for the 16 founders correspond to approximately 1.1M SNPs sites that were called, and the same sites were used for calling the genotypes in the 504 inbred lines. Scott et al. (2021) reported that the variant calls were made unambiguously across the subgenomes for those variants that were included in the dataset. Sites with heterozygous or missing calls in founders were removed from the set of markers, such that the genotype data is fully homozygous. For the inbred lines, the low-coverage sequencing data were augmented using imputation. Heterozygous calls were removed before being input to the STITCH software (Davies et al. 2016). STITCH implements a diploid model, and the performance of imputation is generally dependant on a correct mapping of the loci where the genotypes are predicted. Scott et al. controlled the quality of the imputation with STITCH by comparing the imputed genotypes to a dataset assayed on a microarray for the variants that mapped uniquely to a single subgenome according to stringent BLASTn-filtering. For those array variants, based on the analysis of the patterns in the haplotypes assigned after imputation on the one hand, and on the other hand on the genotype concordance with microarray data, the authors reported a high accuracy in the imputed data. We therefore consider that the imputed data can reasonably represent diploid-like SNP genotypes within the total allohexaploid wheat reference genome. Moreover, the imputation process is expected to correct for moderate sequencing biases such as affinity for a specific allele, by leveraging the identified haplotype mosaic. After selecting a subset of tag SNPs based on linkage disequilibrium (LD), the data for the inbred lines consist in a smaller dataset with the genotypes at ca. 55 K loci over all $3 \times 7$ pairs of chromosomes. Our experiments start out with this 55K SNP dataset, since it would be reasonable to expect a cost-conscious user to select a microarray with a total marker density in this range. While the genotypes used do not represent data from any single existing microarray product, we believe that the careful postprocessing applied to the original sequence data mean that these genotypes accurately reflect the true ones for the curated variants to a high extent. Therefore, the data can be used as the basis for a simulation study. Would an actual microarray be devised based on these SNPs, it would be crucial to ensure that the oligo probes would still hybridize uniquely to a single position within a single subgenome.

We use the founders as the reference panel for imputation, as suggested in Zheng et al. (2018), and the inbred lines as the true data for simulating pooling augmented by imputation in the study population.

The data for the founders and the inbred lines were downloaded from the data repository of UCL (University College London) (London 2021). We use PLINK 1.9 for creating the VCF files from the bim, bed, and fam files. It is known from several studies that the STITCH software can yield still high missing rates after imputation (Nicod et al. 2016; Pook 2021). In practice, the genotypes are set to missing if no posterior genotype probability is larger than 0.9 (Nicod et al. 2016), which is unsuitable for our simulation purposes. After filtering out genetic positions with missing genotypes, both the reference panel and the study population contain the same 20, 572 loci.

#### Genetic map

We download the genetic map provided by INRAE (Unité de Recherche 2018) that uses the reference genome RefSeq v1.0 released by IWGSC (International Wheat Genome 2018). For markers not present in the genetic map, we used a linear interpolation based on the adjacent markers present in the map, as for example suggested in the *1000 Genomes Project* (Pickrell 2015).

### Pooling simulation on full homozygotes in the inbred lines

Relative to diverse human populations, where we have studied pooling strategies previously (Clouard et al. 2022), the individual wheat samples in this study constitute inbred lines. Whether heterozygotes can be ignored in practice will be dependent on the exact nature of the population, but we chose to implement an approach where we assume homozygosity in all loci. Consequently, the decoding is simplified compared to the one presented earlier for human ternary genotypes. The algorithm used for decoding the outcomes of the pooled genotyping experiments with only homozygous genotypes is given in Algorithm 1. The genotypes of the pools are modeled as integers as we assume error-free genotyping. The resolved genotype of any individual is represented as simplex of genotype probabilities for suitability to the imputation.

We keep only $496 = 16 \times 31$ samples randomly selected out of the 504 inbred lines for compatibility with the $4 \times 4$ row-column pooling design which involves 16 samples per pooling block. In an actual pooling scenario, the savings in terms of the number of plates would be dependent on the number of wells per plate. If the study population size has already been adapted to just use a single plate, savings will be limited, but in a typical case the number of plates will be reduced by 50%. If we consider a hypothetical 384-well plate for our 496-individual simulation, individual genotyping would require 2 plates, while pooled genotyping could fit within a single plate. For hypothetical 96-well plates, the full population will use 6 plates, with low occupancy on the final plate. For pooling, 3 plates would be used, with roughly 40% of the wells left empty on the final plate. Each individual genotype at each marker is later retrieved with high accuracy in our pipeline thanks to different methods of statistical inference, with limited computational cost.

### Imputation of genotypes based on pooled tests

The decoding procedure we propose from pooled genotype data is independent of the marker considered, that is by extension decoding is LD-agnostic and does not take into account the actual allelic frequency in the study population. By taking advantage of the genetic structure and characteristics of both the study population and the reference panel, the genotype imputation methods are suitable for enhancing the genotype probabilities inferred from pooling.

We compare two population-based methods, the first one being Beagle 4.1 (Browning and Browning 2016) and the second one being prophaser (Ausmees and Nettelblad 2022). The reasons for using this version of Beagle rather than the most recent release are that we need a method that handles genotype likelihoods (GL) as input.

Both Beagle 4.1 and prophaser can produce genotype posterior probabilities (GP) for any marker, computed using a Hidden Markov Model (HMM). On purpose, no pedigree information is used in our imputation settings. Indeed, while our study uses the founder generation as reference panel, we aim for developing a genotyping method in which imputation can be conducted with any reference panel that could possibly be different from the founders.

There are only 16 homozygous founders in the reference panel, that is only 8 actually different haplotypes, which could impact the accuracy of imputation. However, inherently to the breeding scheme implemented, this limited set of reference haplotypes should cover all existing haplotype variations within the study population.

Both Beagle 4.1 and prophaser were run on chromosome 1A with the corresponding interpolated genetic map. Beagle 4.1 handles the whole study population at once whereas prophaser processes the study individuals one by one. While the genetic map is required for prophaser, it is optional, but recommended, for Beagle 4.1. In practice tough, we have not noticed any significant improvement in the imputation accuracy of Beagle 4.1 when using the map.

Along the Snakemake workflow, we provide examples of scripts that can be used for submitting an imputation job to a Slurm system (Yoo et al. 2003). We underline that these files are specific to the compute cluster we use. They will need adaptations for use on other resources.

#### Imputation with Beagle 4.1

Our Beagle-based imputation approach consists of a first phasing step followed by actual genotype inference. The decoded genotypes are used as initial genotype priors in the HMM. Beagle 4.1 implements a round of phasing iterations preceding the round of imputation iterations. Between these two rounds, an intermediate operation of so-called genotype conforming is required, which consists in making the alleles in the study population consistent with the reference file. The template haplotypes used as hidden states of the HMM in Beagle 4.1 are formed through a model-building step. Model-building consists in merging into clusters the haplotype segments that are identical across the reference and the study samples. This clustering operation decreases the computational complexity of imputation and performs particularly well in large populations with thousands of samples. Beagle 4.1. is multi-threaded, which lowers its execution time with suitable computing resources.

The executable of Beagle 4.1. as well as the documentation was made available for download by Browning and Browning (Browning 2018). For comparison purposes with prophaser, we execute Beagle 4.1. on a compute cluster node with the JVM memory settings -Xss5m. We tested the default settings, as well as explicit changes of the ne and modelscale parameters, one at a time, as suggested in a previous study (Pook 2019).

The node (HP ProLiant SL230s Gen8) has a memory configuration of 128 GiB and consists of 2 CPUs (Intel Xeon E5-2660) with 8 cores each. All the available cores on the nodes are used for execution Beagle 4.1.

#### Imputation with prophaser

With prophaser, the template haplotypes are all the actual haplotypes in the reference panel. By relying on the assumption of coalescence across the population, the imputed haplotypes are expected to be similar to the available templates. The transition and emission probabilities are derived from explicit parameters for the recombination and the mutation rates. We refer to the related publication (Ausmees and Nettelblad 2022) for more details about prophaser.

Because of the small size of the reference panel, we set the effective population Ne size equal to 16. As all the founders are fully homozygous, we enforce the phasing by rewriting the unphased genotypes to a phased state since prophaser requires phased genotypes in the reference panel.

The memory complexity of prophaser scales quadratically in the number of template haplotypes and grows proportionally to the cube root of the number of markers to impute. Given the small size of the reference panel and the small number of markers we use, memory usage should not be an issue in this study. However, larger sizes of populations and sets of markers require that prophaser is run on a large-enough cluster node. Because each of the 496 samples is imputed separately, we prefer executing prophaser on a cluster node for this study as well and we use the same equipment as for running Beagle 4.1. Every study sample can be imputed asynchronously in parallel with the other samples, which reduces the time complexity that would otherwise scale linearly in the number of study samples.

#### Metrics for quantifying accuracy of imputation

For evaluating the performance in accuracy of our approach, we use the genotype concordance on the one hand, and the cross-entropy in the genotype probabilities on the other hand. The details regarding the definitions of these metrics as well as their relevance are presented in our previous research (Clouard et al. 2022). The current study calculates the concordance over all genotypes, including the homozygotes for the reference allele. In some publications, the non-reference discordance rate is given rather than the concordance rate, as we did in our experiment with human data. We choose to complement the concordance metrics with the cross-entropy as this quantity renders better than concordance how close the predictions are to the true genotypes.

In this study, we want to explore to what extent the limited genetic diversity in a wheat breeding context, as well as the homozygosity patterns of inbred lines, affect the accuracy of genotyping through pooling and imputation. Secondly, we would like to verify that a small reference panel can be informative enough for pedigree-free imputation, under the assumption that this panel spans the total haplotype repertoire found in the study population. Last, we expect the rare variants to be accurately genotyped as we found with human data.

## Results

We performed imputation using the founders as reference using both the Beagle and prophaser workflows. We also characterized our marker set and populations in terms of genotype frequencies and other parameters.

### Descriptive population statistics on original data

Our workflow implements to some extent an exploratory analysis of the data for all chromosomes; however, in this manuscript, we present and discuss the results for the chromosome 1A only.

In order to get insights into the original data as well as to specify the effects of simulating pooling on them, we want to identify and describe some genetic characteristics of both the population of founders and the population of inbred lines. These insights might also help to understand the factors affecting the performance of the imputation following pooling.

#### Reference panel of founders

Table 1 gives some statistics describing the genotype data of the 16 founders. As expected from the description of the *Diverse MAGIC Wheat* study, the founders are homozygotes at all loci and no data is missing since the incomplete markers were filtered out when creating the VCF file with PLINK 1.9. The bins 0.05–0.10 and 0.10–0.20 represent nearly $63\%$ of the total number of markers. We note the absence of rare variants in the reference data, which could be explained by the segregation phenomenon that Scott et al. (2021) observed for SNPs with MAF below 0.05 when they compared the 16 founders against resources from the Global germplasm.

**Table 1:** Statistics for markers per MAF bin in the population of founders

	0.05–0.10	0.10–0.20	0.20–0.30	0.30–0.40	0.40–0.50	Total
Counts	333	401	151	184	101	1170
Proportions^a^	0.285	0.343	0.129	0.157	0.086	1.000
% missing genotypes	0.000	0.000	0.000	0.000	0.000	0.000
% heterozygotes^b^	0.000	0.000	0.000	0.000	0.000	0.000

The bin [0.00, 0.05] is not shown since there are no variants in this range. Indeed, the minimal value for MAF is $1/16 = 0.625$ in the case only one copy of the minor allele is found among the founders

^a^SNPs proportions per MAF bin with respect to the total number of SNPs on the genetic map

^b^The reason for the absence of any heterozygote lies in the filtering operations that were applied to the data by Scott et al. (2021). For the founders, the sites with heterozygous calls in a any founder were excluded from the set of SNPs

#### Study population of inbred lines

Table 2 indicates that all study samples are full homozygotes as the reference samples are, which was expected since in the dataset we use for our simulations, the heterozygous genotypes were replaced with missing entries and imputed with STITCH.

**Table 2:** Statistics for markers per MAF bin in the population of inbred lines (true and pooled data)

	0.00–0.05	0.05–0.10	0.10–0.20	0.20–0.30	0.30–0.40	0.40–0.50	Total
Counts	74	266	359	223	140	108	1170
Proportions^a^	0.063	0.227	0.307	0.191	0.120	0.092	1.000
% missing genotypes (true data)^b^	0.000	0.000	0.000	0.000	0.000	0.000	0.000
% heterozygotes (true data)^b^	0.000	0.000	0.000	0.000	0.000	0.000	0.000
% missing genotypes (pooled data)	2.558	7.598	26.307	48.606	66.228	75.196	34.092
% heterozygotes (pooled data)^c^	0.000	0.000	0.000	0.000	0.000	0.000	0.000

^a^SNPs proportions per MAF bin with respect to the total number of SNPs on the genetic map

^b^ Scott et al. (2021) intentionally set to missing sites with heterozygous calls and imputed all missing entries with STITCH, such that the data is fully homozygous

^c^The decoding algorithm we use assumes there are no heterozygous sites

Moreover, the inbred lines are the last generation in the MAGIC scheme and thus derive from several cycles of selfing, which should in practice ensure that all plants are fully homozygous. Overall, the distribution of the true genotypes in the inbred lines is somewhat different than the one in the founder population, although the bins 0.05–0.10 and 0.10–0.20 remain the largest ones. The presence of low-frequency variants could be explained by a phenomenon of genetic drift through the breeding process. Such dissimilarities in the distribution of genotypes could impact the accuracy of imputation. However, in the case of Beagle, ca. 30 times more study samples than founder individuals are used when computing the template haplotypes. The differences might therefore be mitigated and only have an insignificant effect.

**Fig. 2:**
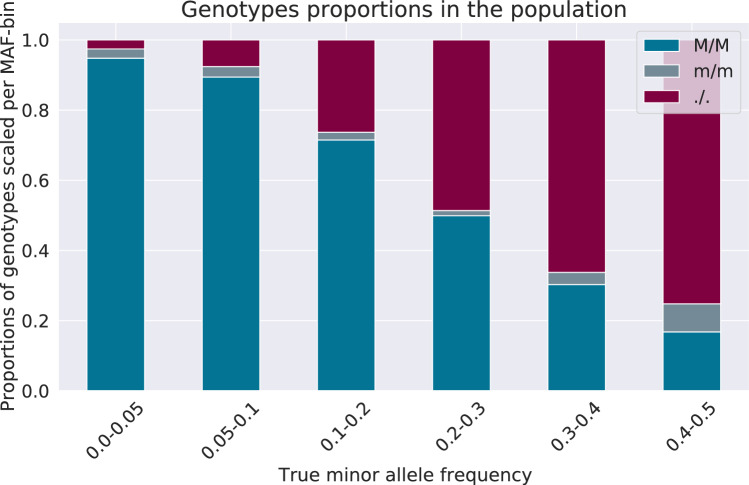
Genotype proportions in 496 inbred lines (pooled data) for 1170 SNPs on the chromosome 1A Proportions scaled and given per bin. M is the major allele and m the minor one. ’./.’ indicates that the genotype is not decoded from pooling, i.e., missing. Thanks to the binary nature of the genotypes in a fully homozygous population, there are no cases where one allele only can be resolved while the other is ambiguous. The genotypes are either fully missing or fully known

Figure 2 shows the proportions of the reference and alternate homozygous loci per MAF bin across the pooled genotypes of the inbred lines. Inherently to the shortages of a pooling strategy and the pooling design used, some genotypes cannot be resolved from the pools, especially when both alleles at the locus are observed in at least $10\%$ of the samples. The percentage of missing data increases with the MAF and reaches on average $75\%$ for the loci with the highest MAF. Over all markers, around a third of the genotypes are not decoded after the pooling simulation, which is a low rate in the context of imputation where some methods can handle up to $90\%$ missing data. Nonetheless, we have earlier demonstrated (Clouard et al. 2022) that the non-randomness of the distribution of the missing data from pooling poses particular challenges to the imputation algorithms.

**Table 3:** Performance of Beagle 4.1 for various sets of parameters

Parameters	Accuracy^a^		Computational performance^b^	
	Concordance	Cross-entropy	Running time (hh:mm:ss)	Memory usage (maximum in GiB)
all default	0.86152	1.54605	02:28:08	10.7
**modelscale=1.5,** **other default**	**0**.**86494**	**1**.**51749**	**00:02:02**	**0**.**7**
modelscale=2.0, other default	0.85770	1.59198	00:01:03	0.7
ne=16, other default	0.86309	1.56898	02:30:10	7.5
ne=5000, other default	0.86147	1.55936	02:33:09	9.3
modelscale=1.5, ne=5000, other default	0.86328	1.53511	00:01:16	0.7
modelscale=1.5, ne=16, other default	0.86277	1.57283	00:03:48	0.7

Bold font highlights the settings achieving the best performance. The computational performance is given for processing all the 496 study samples together

^a^The accuracy is calculated as the average concordance or cross-entropy over all markers, regardless of the MAF

^b^Computations run on 1 node with dual CPUs (Intel Xeon E5-2660), 8 cores per CPU, that is 16 cores in total and 128 Gigabyte RAM

**Table 4:** Distribution of the markers genotyped after pooling and after imputation per data MAF bin

	MAF bin Imputation method^b^	0.00–0.05	0.05–0.10	0.10–0.20	0.20–0.30	0.30–0.40	0.40–0.50
Fully decoded before imputation	Beagle 4.1						
	Prophaser	70.036	249.143	267.109	115.569	46.651	26.716
Exact matches after imputation	Beagle 4.1	71.093	261.877	324.651	176.222	99.956	79.401
	Prophaser	71.393	265.020	343.740	205.004	125.448	95.667
% heterozygotes after imputation	Beagle 4.1						
	Prophaser	0.000	0.000	0.000	0.000	0.000	0.000
Proportion of fully decoded before imputation^a^	Beagle 4.1						
	Prophaser	0.973	0.923	0.742	0.523	0.336	0.247
Proportion of exact matches after imputation^a^	Beagle 4.1	0.987	0.970	0.902	0.797	0.719	0.735
	Prophaser	0.992	0.982	0.955	0.928	0.903	0.886

^a^SNPs proportions per MAF bin with respect to the number of SNPs in the bin

^b^Beagle 4.1: modelscale=1.5, other parameters to default

We observe in Table 4 that a large share of the pooled genotypes can be successfully decoded. This could be partly explained by the binary nature of the genotype data in the NDM wheat population, to the difference of human genotypes that are ternary. For instance, $74.2\%$ of the genotypes of markers with a MAF in 0.1–0.2 are fully decoded in wheat whereas this percentage was only $47.2\%$ in human.

**Fig. 3:**
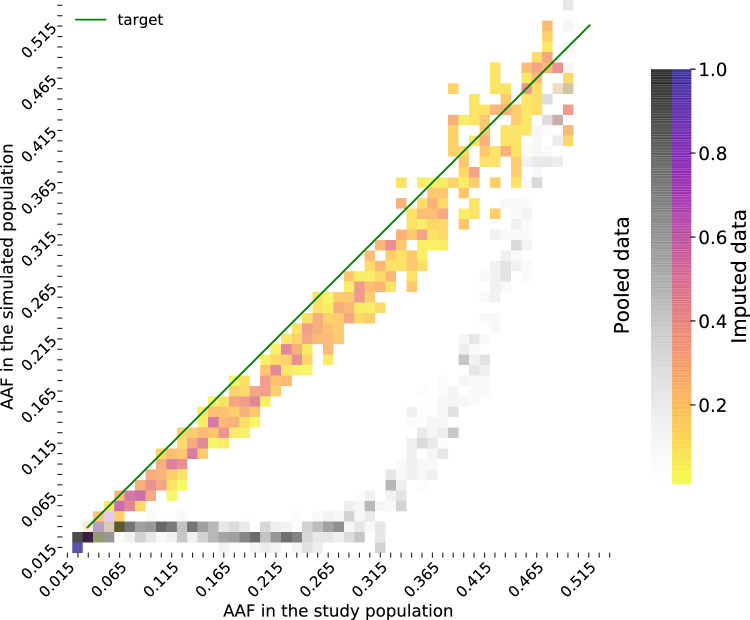
Proportions of markers per AAF-bin in the pooled and the imputed study population with respect to the AAF-bin in the true study population The simulated population corresponds on the one hand to pooled and decoded study population (gray shades), on the other hand to the pooled and imputed study population with prophaser (bright shades). The target line stands for the ideal situation where there is an exact correlation between the AAF of markers in the true study population and in the simulated population. The darkest color shades on the heatmap close to the origin of the plot are close to the target line, which denotes that a high proportion of low-AAF markers (AAF below $5\%$) is decoded and imputed at a correct frequency. The yellow shades indicate on the contrary a lower concentration of markers and the increased spreading of the colored cells suggests that imputation accuracy has a higher variance for variants with $MAF > 0.3$

The heatmap displayed in Fig. 3 provides further insights into the impact of successive pooling simulation and imputation on the observed allelic frequencies in the inbred lines. After decoding the pooled outcomes only, we can notice that the frequency of the alternate allele is largely underestimated at most loci for which the true AAF is in the range 0.05–0.45.

The lowest AAF values (up to ca. 5%) correspond to situations where either the large majority of the samples is homozygous for the reference allele at a given locus, and most of the pools are therefore homogeneous, which enables full decoding. When the true AAF is in the range 0.05–0.30, a few homozygotes for the alternate allele might be found in the same pooling block and can thus not be decoded. This biases the proportion of observable genotypes against the homozygotes for the alternate allele. For larger AAF values, the proportions of both homozygotes are closer, such that most of the pools are heterogeneous, and very few samples can be decoded as the lighter gray shades indicate. However, the prophaser imputation method manages to greatly correct this bias in the allelic distribution despite a slight residual underestimation overall. This heatmap makes the caveats of pooling more obvious and also motivates the implementation of an adequate imputation step for complementing pooling and by that taking full advantage of this cost-cutting strategy.

### Performance of imputation

Table 4 lets us verify that both Beagle 4.1 and prophaser can impute all the genotypes that are unresolved after pooling since there is no missing data. That is, we manage to estimate the genotypes at 1170 loci in 496 samples based on the simulated outcome of array-based genotyping for only 248 pools. Furthermore, no heterozygous genotypes are predicted, which is a relevant result given the absence of heterozygotes in the true population. We underline that the initial LD pruning applied for creating the 55K dataset makes the imputation task in our pipeline relatively challenging since the markers with high LD which would likely be correctly imputed after our pooling simulation are filtered out.

#### Beagle 4.1

Table 3 presents the results for the computational performance and the accuracy of Beagle 4.1 for various sets of parameters. We use as a baseline for comparison the values measured with full default settings. On the whole, we find that modifying only the model scale default value to 1.5 and processing the entire population at once yields the best trade-off in performance, that is an increased accuracy up to $86.5\%$ concordance and a much higher computational efficiency. Increasing the modelscale parameter might have a strong impact on the computational performance because a larger model scale lets less similar haplotypes be clustered together in the model-building step of Beagle (Pook 2019). As a result, the clustered tree defines fewer template haplotypes which in its turn speeds up the computations with the imputation HMM as well as uses less memory. Modifying the default parameters modelscale or ne seems to have only little effect on the accuracy of imputation. We also executed a sample-wise imputation with Beagle 4.1 (modelscale=1.5 and other parameters to default) but since the accuracy was not improved we do not present the results.

**Fig. 4:**
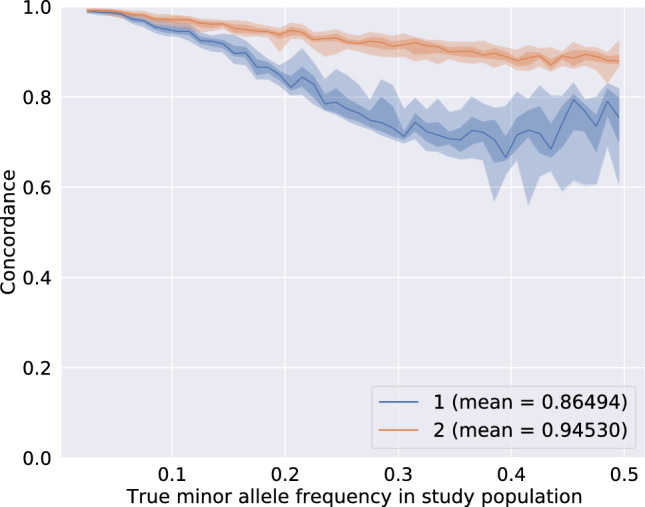
Genotype concordance for Beagle 4.1 (1) and prophaser (2) The central line is the median and the shadowed areas delimit the quantiles 0.0, 0.01, 0.25, 0.75, 0.99, 1.0. The x-axis was built from 0.05-long MAF bins within which each marker concordance score was computed as the mean score in a five-marker-long window including the 2 previous and 2 next markers sorted per ascending MAF. The maximum score for perfect concordance is 1.0

**Fig. 5:**
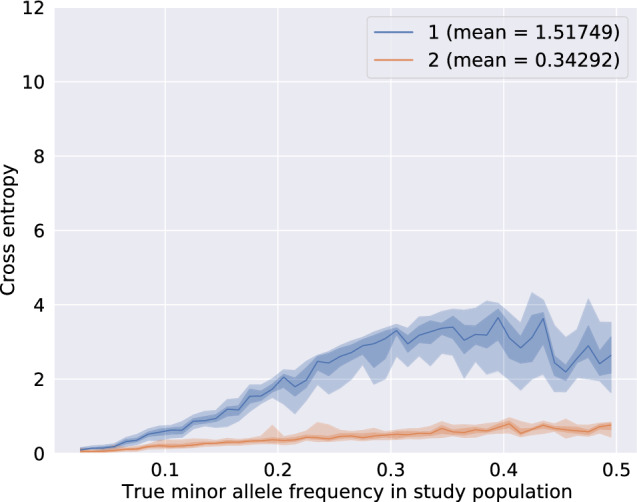
Genotype cross-entropy for Beagle 4.1 (1) and prophaser (2) The central line is the median and the shadowed areas delimit the quantiles 0.0, 0.01, 0.25, 0.75, 0.99, 1.0. The x-axis was built from 0.05-long MAF bins within which each marker concordance score was computed as the mean score in a five-marker-long window including the 2 previous and 2 next markers sorted per ascending MAF. The best cross-entropy score is 0.0 and indicates that the predictions exactly match the true genotype probabilities. Higher scores are caused by larger differences between the predicted and the true genotype probabilities

Figures 4 and 5 compare the accuracy of imputation of Beagle 4.1 with the best parameter settings against prophaser. The accuracy of imputation is especially high for variants with rare alternate alleles (top left of the plot), which are usually a caveat of the imputation methods. Overall, Beagle 4.1 achieves a concordance score of $86.5\%$ and a cross-entropy score of 1.517. While the model implemented by Beagle has a clear advantage in computational performance, it seems to struggle with making use of the tailored genotype probabilities resolved from pooling. The node merging rules might not be well-suited for dealing with data that is not missing at random as it is the case with pooled genotypes. Moreover, the irregular shape of the quantile envelopes and the uneven median line suggests that Beagle 4.1 produces heterogeneous results for markers having close MAF. This might be also due to the node merging step that over- or undersamples some haplotypes when creating the templates.

#### Prophaser

Figures 4 and 5 illustrate that prophaser achieves a nearly completely exact imputation of very low-MAF markers ($MAF < 0.08$). The overall average concordance resp. cross-entropy reaches $94.5\%$ resp. 0.343, but the accuracy decreases as MAF increases.

The low cross-entropy value renders that the genotype predictions are very close to the true value. This could be explained by the low occurrence of cases in which very low probabilities are assigned to the actually correct genotypes, as if the model implemented in prophaser would have the ability to sense ambiguity when resolving the genotypes. For instance, at the genetic position 9,234,368, we find a sample whose true genotype is homozygote for the alternate allele, that is the genotype probabilities are represented by the tuple (0, 0, 1). After pooling simulation and imputation, Beagle predicted the genotype probabilities (1, 0, 0) and prophaser predicted (0.407351, 0, 0.592649). That is, the best-guess prediction of Beagle is homozygote for the reference allele with probability 1, which is fully incorrect, and the cross-entropy is thus maximal in this case. The best-guess prediction prophaser is homozygote for the reference (resp. alternate) allele with probability 0.59 (resp. 0.41), which is weakly correct, and the cross-entropy is submaximal but not null. The narrow quantile envelopes both for the concordance and the cross-entropy indicate that accuracy is highly consistent over markers with similar MAF values. The counts of exact matches presented in Table 4 confirm the observations made in the figures. The markers having $MAF < 0.1$ are on average imputed exactly in $98.2\%$ of the cases and even up to $99.2\%$ when MAF drops below 0.05. While it is true that most of these low-MAF markers are decoded from the pools at up to $97.3\%$, increasing the percentage of the matches by 2 points is a relevant improvement, since it is crucial in order to actually pinpoint the identity of the carriers of the rare variant.

### Computational performance

The execution of all the steps in the workflow that precede imputation, that is from the initial downloading of the genotype data until the computation of the interpolated maps, takes about 3 min and 20 s and requires at most 1.1 GiB memory on a compute node whose characteristics are described in the methods for imputation.

The computations with prophaser for imputing the unresolved genotypes take around 475 milliseconds per study sample and require an insignificant amount of memory. For Beagle, the best results are achieved by processing the entire study population at once for imputing all samples in 2 min and using 0.7 GiB memory.

## Discussion

Since our approach of combining pooling with imputation is novel, there are few points of direct comparison in the literature. Our best score for average concordance in the imputed data is about $94.5\%$, which has the same order of magnitude as imputation from two-way-pooled GBS data that was sequenced at 0.5x coverage (Technow and Gerke 2017). Concordance was not binned by MAF in that study, which we think is crucial in order to understand the performance of a method. Pook (2019) included a figure rendering the error with respect to the allele frequency, but this was in a scenario with specific genotypes missing at random, rather than the structured frequency-dependent missingness patterns arising from pooling, and as such resulting in a very different overall trend. The error is indeed the lowest for common variants and the lowest for the rare ones, while the complementing nature of pooling and imputation gives our results the opposite overall trend.

Overall, we obtain high genotyping accuracy, especially with regard to low-frequency alleles. The combination of computer-assisted decoding and genotype imputation is motivated by the interest in saving costs for genotyping. While we treat the samples in both the study population of inbred lines and the reference panel of founders as they would be unrelated, the very good accuracy of imputation achieved with prophaser may be explained by the actual relatedness inherent to the breeding scheme, such that every inbred line is a perfect mosaic of the founders both in practice and in the imputations model. We see room for improvement and further investigations of this pooling strategy.

The challenge of large-scale genotyping is represented through two main dimensions which are the number of markers to genotype for one thing, and the number of samples to analyze in the target population. Conventional genotype imputation from low-density to high-density sets of markers has long been a powerful computational tool for reducing the cost of genotyping by only testing the majority of samples on lower density chips. This results in the need for smaller and thus less expensive microarrays. Our pooling strategy instead acts in the other dimension, by cutting the number of samples to test on the microarrays, without decreasing the marker density. In practice, to maximize the savings, one should try to fill all wells in the plates used. Since the number of wells needed for a population of a certain size is cut in half, workflows might need adaptation to allow for pooling into blocks and then filling each plate. Assuming working with 96-well plates, this implies that we should aim for genotyping a number of pools which is a multiple of 96 in order to not leave any empty well, or in other words, a total sample count of $96 \times 2 = 192$ individuals is the minimum optimal size of population given the filling constraint (assuming no wells are reserved for controls). In this regard, our simulation is not optimized for the laboratory since it would require two fully filled plates and one filled with only 56 pooled samples. On the computational side, the complexity in time of prophaser is linear in the number of samples, but overall, our pipeline is highly parallelizable, per block for pooling, and per individual for imputation.

We are aware our simulations present a weakness since they assume an error-free genotyping, which is a situation that is not encountered in practice. It would be relevant and ideal to apply our approach to the data of actual pools that were though not available to us when we conducted this study. However, in preliminary experiments, we explored imputation based on pooled data in which we added artificial noise with varying intensity for modeling possible genotyping inaccuracies (unpublished research). We observed that reasonable levels of noise do not significantly degrade the accuracy of imputation. Thus, we believe that an actual workflow of pooling followed by imputation using, e.g., prophaser would also be robust to noise, assuming that the calling of individual pooled genotypes took the skewed allele proportions in samples into account, in a way similar to what is already done for polyploidic markers (Clevenger et al. 2015; Blischak et al. 2017).

In our previous simulation study of pooling on human data, we implemented an iterative approach for the decoding of the pools, especially in order to give reasonable prior estimates to imputation of heterozygote vs. homozygote alternate allele genotype probabilities. This was less necessary for inbred data. However, we believe that a more well-developed approach for information sharing within blocks, taking preliminary imputation results into account, could be worthwhile. The benefits of such an approach are probably more pronounced for the structured and highly related populations frequently found in plant breeding.

While the time and the memory requirements for imputation are negligible and do not represent a bottleneck in our study case, the computational efficiency of prophaser would be critical for performance if larger populations and sets of markers are investigated. As the founders and the inbred lines are homozygous, it would be possible to use an explicitly haploid HMM, similar to the one proposed by Thorn et al. (2021).

Beyond the need for testing our approach on real and likely noisy data collected from actual pools, investing various other datasets could reveal weaknesses and strengths of the pooling strategy. For instance, scaling up this study could facilitate the comparison between Beagle 4.1 and prophaser regarding the computation time and the memory resources used. The set of markers we used for our simulation purposes is particularly sparse due to strong filtering and LD pruning implemented for the tag SNP dataset in Scott et al. (2021). We expect our approach to achieve above $94.5\%$ accuracy with denser SNP sets that are also likely to be more rich in rare variants. We believe that the accuracy would remain high with larger or smaller populations, as long as all individuals have a similar genetic structure, such that the 16 founders we use suffice for describing this structure. For example, enlarging the study population with samples of north-west European origin should not degrade the accuracy since Scott et al. (2021) found that the 16 founders from the UK are representative of the germplasm found in this area. Imputation with other reference panels, such as cultivars from a different geographical origin, a subset of the inbred lines, or a mix of founders and inbred progeny, might elucidate to what extent the composition and size of the reference panel can impact imputation accuracy. Overall, we believe that existing results on imputation quality in classical imputation and the relevance of an appropriate reference panel would translate to the pooling imputation setting. Larger pool sizes would decrease the cost for testing equipment even more and let us evaluate the trade-off between cost-effectiveness in laboratory testing and the accuracy of genotyping.

We have been considering adapting the method of combined use of pooling and imputation for handling GBS data as suggested by Technow and Gerke (2017). This type of data is known to be more noisy than array-based genotype data though; therefore, an additional step of denoising or error correction may be needed in our workflow in order to handle the increased uncertainty of the genotype data. The expense for library preparation is the most impacting component of the cost in sequencing experiments. If one would do library preparation and sequencing only after first pooling the samples, a pooling strategy could therefore contribute to decrease the cost of sequencing and genotyping with GBS as well, still augmented by imputation.

## Conclusion

This study suggests that a pooling strategy can be successfully implemented for genotyping SNPs at reduced cost in a MAGIC wheat population. Given the characteristics and quality of the dataset we use as basis for our simulations, we propose to model pooled array data from them and experiment our strategy with the simulated pools. The strategy consists in combining a probabilistic pattern-consistent resolution of the pools and genotype imputation. The genotyping accuracy from pooled data was the highest with a coalescent model, which performed better than the haplotype clustering model implemented in Beagle.

Despite the small reference panel used and the differences in allelic frequencies with the study population, we obtain high genotype concordance and low cross-entropy after pooling and imputation, even without pedigree information. Pooling benefits the low-frequency variants the most, as it was the case with human data, even with a simpler algorithm for resolving the pools.

Being able to achieve high accuracy with reference panels of small size is advantageous with regard to the computational cost and the expenses for genotyping the reference panel are lower. Moreover, it might be possible to augment the size of the study population (inbred lines) without the need for enlarging the panel of founders or sacrificing the accuracy of imputation.

With the pooling design we investigate, we test in each pooling block the genotype of 8 pools instead of 16 single samples and therefore achieve a cost reduction in $50\%$ for the microarray testing equipment. We observe a better resolution of the pools in wheat data compared to human data, which could be explained by the full homozygosity in the population of inbred lines, decreasing the level of residual ambiguity after pool decoding.

Based on the highly successful simulation results, we believe that it would be worthwhile to explore the true challenges of applying the approach to real array data with the added challenges from conducting pooling in practice.

## Algorithms

Algorithm 1 describes the deterministic rules for resolving the pools of the row-column design into individual genotypes. The decoding procedure assumes that any individual *i* is homozygous at any loci *j*, such that the outcomes are genotypes that are either homozygous or completely missing.

**Algorithm 1:**
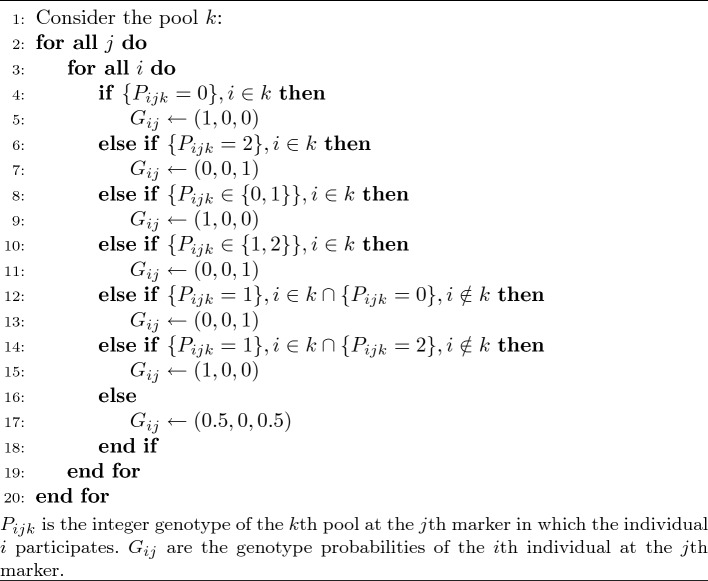
Homozygous genotype decoding with a NORB pooling design

## Supplementary information

The simulations conducted can be reproduced via a workflow which can be found at https://github.com/camcl/poolimputeSNPs. This workflow uses source codes that are accessible at https://github.com/camcl/genotypooler/tree/magicwheat (*genotypooler*) and at https://github.com/scicompuu/prophaser/tree/multilevel (*prophaser*).

## Data Availability

The datasets supporting the conclusions of this article are available at http://mtweb.cs.ucl.ac.uk/mus/www/MAGICdiverse/MAGIC diverse FILES/ (genotype data) and https://urgi.versailles.inra.fr/download/iwgsc/IWGSC RefSeq Annotations/v1.0/iwgsc refseqv1.0 recombination rate analysis.zip (genetic maps).

## Abbreviations

AAF: Alternate allele frequency
AIL: Advanced intercross lines
GBS: Genotyping by sequencing
GS: Genomic selection
GWAS: Genome-wide association studies
IWGSC: International Wheat Genome Sequencing Consortium
LD: Linkage disequilibrium
MAGIC: Multi-parent advanced generation intercross
MAS: Marker-assisted selection
NIAB: National Institute for Applied Botany
NDM: NIAB Diverse MAGIC
NGS: Next-generation sequencing
QTL: Quantitative trait locus
RIL: Recombinant inbred lines
SD: Segregation distortion
SLURM: Simple Linux Utility for Resource Management
SNP: Single nucleotide polymorphism
